# ChatGPT has the potential to enhance antiretroviral therapy adherence among adolescents with HIV in sub-Saharan Africa

**DOI:** 10.1080/10872981.2023.2246781

**Published:** 2023-08-10

**Authors:** Samuel Kizito

**Affiliations:** International Center for Child Health and Development, Brown School, Washington University, St. Louis, MO, USA

To the editor,

Artificial intelligence has a role to play in the fight against HIV. Globally, 1.7 million adolescents live with HIV, of whom 89% live in sub-Saharan Africa (SSA) [1]. Good adherence to antiretroviral therapy (ART) results in prolonged survival and reduced HIV transmission [2]. However, factors like stigma, low HIV knowledge, and poor healthcare infrastructure have undermined ART adherence – especially in SSA [3,4].

The language model ChatGPT has taken the world by storm with its impressive conversational abilities and has rapidly become a widely used tool [5]. ChatGPT can engage in human-like interactions, providing an opportunity for personalized and discreet advice [6]. Its potential application in HIV care has been recognized as a significant breakthrough and is expected to enhance healthcare quality globally [7]. The use of AI in improving ART adherence among adolescents, particularly in resource-constrained settings, requires special attention.

ChatGPT’s full-time availability and real-time responses make it an invaluable tool for patients seeking information about ART. It can dispel myths and provide evidence-based information to help patients make informed decisions about their treatment [8]. In contexts where privacy is a concern, an AI-driven conversation provides anonymity, potentially encouraging open dialogue about ART adherence and related challenges. In SSA, where healthcare resources are stretched thin, AI becomes even more critical, bridging the gap by providing continuous support and guidance to ALHIV.

Although ChatGPT possesses the potential to revolutionize HIV management, its effectiveness relies on streamlining particular challenges [8]. Among these are limited smartphone access, inconsistent power supply to charge devices, and unstable internet access [9]. These hurdles can undermine the potential of ChatGPT in SSA, where these challenges are prevalent. It is essential to prioritize strategies that bolster digital infrastructure and make affordable data packages available. Finally, AI is prone to errors and may occasionally need more accuracy; hence should not be used to replace human healthcare providers but to complement their expertise and enhance patient care, and ALHIV needs to be advised about when to seek additional help.

In conclusion, AI can be an excellent tool for providing information and support and should be used as a supplementary tool that enhances the existing healthcare infrastructure and resources. Incorporating conversational AI into HIV management can provide innovative avenues to improve ART adherence among ALHIV in SSA. However, the maximal success of this approach is contingent upon addressing underlying challenges through careful planning, rigorous research, and a commitment to improving digital infrastructure.
